# Protective Effects of Vitamin E and Selenium Nanoparticles Against Mercury Induced Growth, Blood and Histopathological Changes in Tilapia, *Oreochromis niloticus*

**DOI:** 10.1002/vms3.71305

**Published:** 2026-11-03

**Authors:** Mir Ashrafia Ananna, Abhijit Dash, AKM Afzal Hossain, Md Ruhul Amin, Md Shahjahan, Md Al‐Emran

**Affiliations:** ^1^ Laboratory of Fish Ecophysiology Department of Fisheries Management Bangladesh Agricultural University Mymensingh Bangladesh

**Keywords:** histopathology, mercury, mitigation, selenium, tilapia, vitamin E

## Abstract

**Introduction:**

Heavy metal pollution, especially from mercury (Hg), poses a significant risk to the aquatic organisms. The present study examined the effectiveness of dietary selenium nanoparticles (SeNP) or vitamin E in mitigating mercury‐induced physiological effects including growth and feed utilisation, blood biochemical indices, histopathological changes in gills and intestine in Nile tilapia, *Oreochromis niloticus*.

**Materials and Methods:**

After 15 days of acclimation, 20 fingerlings of tilapia were released in each glass aquaria having four conditions such as control (free from test chemical/additives), mercury (Hg; 0.03 mg/L), Hg + Se (Hg; 0.03 mg/L, and selenium; 3 mg/kg feed) and Hg + Vit‐E (Hg; 0.03 mg/L, and vitamin E; 100 mg/kg feed) with three replicates for each treatment. The trial was lasted for 42 days. After the culture period, the fish were sampled and growth, feed conversion, blood profiles (i.e., glucose and haemoglobin), blood cytology (i.e., erythrocytic cellular and nuclear structures) and histopathology of gills and intestine were evaluated.

**Results:**

The present study finds that chronic exposure to mercury significantly (*p < *0.05) reduced growth, altered blood biochemistry (i.e., increased glucose and haemoglobin), enhanced erythrocyte cellular and nuclear deformities and significantly higher pathologies in histology of gills and intestinal tissues. However, the current study recorded significant mitigation of growth inhibition, restoration of blood profiles and improvement of histological lesions following the incorporation of SeNP or vitamin E into the basal diet. Supplemented group experienced significantly better WG, SGR, FCR and survival, moreover blood glucose, haemoglobin and erythrocytic perturbations were normalised along with a lesser frequency of lesions in gills and intestine, suggesting significant restoration of physiological damage instigated by mercury exposure.

**Conclusion:**

Therefore, these findings can be an effective tool in managing heavy metal‐induced toxicity resulting physiological damage in cultured fishes, including tilapia.

## Graphical abstract

• Mercury caused significant loss in growth and feed conversion efficiency, altered blood profiles and increased gill and intestinal pathologies of tilapia.

• Vitamin E and Selenium nanoparticles supplementations significantly improved growth and feed conversion efficiency, blood profiles and gill and intestinal pathologies of tilapia.

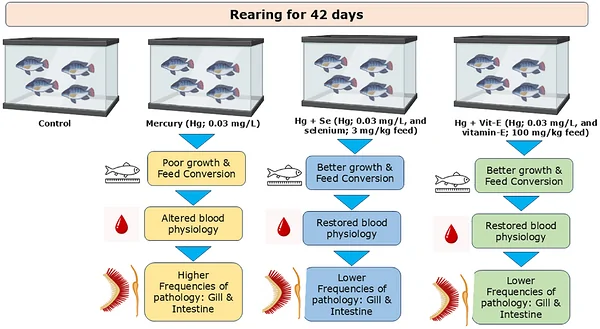

## 1 Introduction

Mercury (Hg) is a ubiquitous environmental contaminant released from both natural and anthropogenic sources that undergoes complex biogeochemical transformations in aquatic systems and eventually concentrates within food webs (Harding et al. 2018). In aquatic organisms including fish, mercury can produce multi‐system toxicity that manifests as reduced growth, altered metabolism, impaired osmoregulation, haematological disturbances and marked histological damage to gill, liver and kidney tissues (Canham et al. 2020). The toxic potency of mercury in fish is mediated by its chemical forms and bioavailability. Moreover, inorganic mercury can be methylated by sediment microorganisms to form methylmercury, the organic species that bioaccumulates and biomagnifies through trophic networks and poses the greatest risk to consumers including fish (Zhang et al. 2010). Mechanically mercury exposure disrupts redox homeostasis, depletes cellular antioxidants such as reduced glutathione, inhibits selenoenzymes and provokes lipid, protein and DNA oxidation; these processes trigger observed decline in growth performance, impairment of blood parameters and structural tissue lesions reported in several fishes (Canham et al. 2020; Zulkipli et al. 2021). Supplements that restore antioxidant capacity have been proposed to mitigate mercury toxicity in fish including nutritional antioxidants. Vitamin E functions as a lipophilic radical scavenger that protects membrane lipids and preserves cellular integrity (Moniruzzaman et al. 2017).

Selenium (Se) is an essential trace element crucial for better performance and wellbeing of aquaculture species which can protect the fish from cell oxidation caused by several stressors and toxins including high density, transportation, poor water quality, neurodegeneration, cardiovascular and immune dysfunction (Kumar et al. 2018), and infectious disease by releasing antioxidants and thyroid hormones (Kumar et al. 2024). Mercury (Hg) depresses antioxidant enzymes such as SOD, CAT and GPx and increase lipid peroxidation in liver, however, dietary selenium has been reported to restore these enzymes (Cabezas‐Sanchez et al. 2019; Kothari and Choughule 2015; Shinde et al. 2025). Research suggests that selenium binds with mercury and forms selenoprotein complexes which are comparatively less bioavailable and reduce tissue concentration of mercury (Manceau et al. 2021; El Hanafi et al. 2024). In contrast, low selenium status increases mercury susceptibility, while adequate selenium can reduce mercury‐induced biochemical and developmental damage (Ganther 1980; da S. Cabral et al. 2025). Recent studies have examined selenium nanoparticles exhibiting high bioavailability, lower acute toxicity compared with some inorganic forms, and promising effects on growth, antioxidant enzyme activity and immune indices in cultured fishes (Dawit Moges et al. 2022; Ahmed et al. 2025).

Vitamin E (α‐ tocopherol) is a fat‐soluble, chain‐breaking antioxidant that scavenges lipid peroxyl radicals and prevents propagations of lipid peroxidation (El‐Sayed and Izquierdo 2022; Hamre 2011). Moreover, vitamin E helps to maintain the functional integrity of membrane‐bound antioxidant enzymes such as SOD, CAT, GPx and decrease the need for compensatory upregulation under oxidative stress (El‐Sayed and Izquierdo 2022; Tocher et al. 2002). Studies indicate that vitamin E and selenium may act synergistically to diminish tissue mercury accumulation and to ameliorate physiological and histological alterations induced by mercury and methylmercury in fish models. However, the protective efficacy of selenium nanoparticles alone or vitamin E has not been comprehensively evaluated against inorganic mercury exposures in tilapia.

Growth and feed utilisation indices (i.e., weight gain (WG), specific growth rate (SGR) and feed conversion rate) indicate overall organismal performance in aquaculture fishes (Ferdous et al. 2024). Hematobiochemical parameters (i.e., glucose and haemoglobin) reflect systemic stress marker and oxygen transport (Shahjahan et al. 2022). Blood cytological markers such as the morphology of erythrocyte and their nucleuses provide important insight on chromosomal and nuclear damage (Shahjahan et al. 2022; Shahjahan et al. 2018; Ashaf‐Ud‐Doulah et al. 2019). Histopathological changes of gill and intestine documents tissue‐level lesions underlying functional decline (Kumar and Sahu 2024; Hossain et al. 2022).

Nile tilapia, *Oreochromis niloticus*, is a globally recognised aquaculture species that is both vulnerable to environmental contaminants and a principal source of animal protein for humans (Amin et al. 2025; Debnath et al. 2023). Despite this species having immense aquaculture significance, species‐specific studies on the mitigation of mercury‐induced alterations by selenium nanoparticles (SeNP) or vitamin E still remain limited. Besides, these nutritional mitigation strategies for mercury toxicity have both animal health and food safety implications (Brodziak‐Dopierała and Fischer 2023). Therefore, the current study tests the hypothesis that dietary vitamin E and selenium nanoparticles confer protection against sublethal mercuric chloride exposure in Nile tilapia as evaluated by growth performances, haematological indices and histopathological outcomes. Results are intended to clarify the potential of combined antioxidant strategies to reduce mercury‐induced physiological impairment in aquaculture species.

## 2 Materials and Methods

### 2.1 Chemicals and Reagents

Mercuric chloride (HgCl_2_, 271.52 gm/mol, analytical grade, Sigma–Aldrich, USA), sodium selenite (Na_2_SeO_3_, 172.94 gm/mol, analytical grade, Sigma–Aldrich, USA) and DL‐α‐Tocopherol acetate (>99% purity, ACI Pharmaceuticals Ltd., Bangladesh) were used as the experimental sources of mercury, selenium and vitamin E, respectively. Reagents were stored and handled according to the manufacturer's recommendations, and stock solutions and supplemented feeds were prepared fresh prior to use.

### 2.2 Preparation of Stock Solution and Feed Formulation

To prepare a stock solution of 1 mg/mL Hg, 1.35 gm HgCl_2_ was dissolved in 1000 mL of distilled water, as mercuric chloride (HgCl_2_) has a molecular weight of 271 g/mol, containing 200 gm of mercury per mole. For the selenium‐supplemented feed, a dose of 3 mg/kg feed was achieved by mixing 6.57 mg of sodium selenite (Na_2_SeO_3_) with per kg commercial diet using rice gruel (1/10^th^ of the total feed) as a natural binder. Similarly, vitamin E‐enriched feed was prepared by incorporating 106 µL of DL‐α‐Tocopherol acetate (99% purity) in per kg commercial feed, using corn oil (1/10^th^ of the total feed weight) as a binder. The coated feed was thoroughly mixed to ensure uniform distribution and air‐dried in a cool place. Finally, the stock solution of mercury was stored in a dark container to prevent light‐induced degradation, while the coated feeds were kept in a cool place to preserve their stability and extend shelf life.

In this study, Hg was applied at 0.03 mg/L, corresponding to 1/10th of the 96 h LC_50_ value for Nile tilapia as reported by Jasim et al. (2016). This methodological framework is commonly used to evaluate early physiological impairments under toxicant exposure. The selected concentration of Hg aimed to induce sublethal but biologically significant stress, which allowed us to assess the effectiveness of mitigation approaches without causing acute lethality. Furthermore, the doses of selenium nanoparticles (3 mg/kg) and vitamin E (106 µL) were selected from previous studies, in which the authors showed that such dietary supplementation significantly improved growth performance and overall physiology in Nile tilapia (Rohani et al. 2023). Although these supplementation levels were not specifically optimised for mercury exposure conditions, we hypothesised that these supplementation levels may alleviate Hg‐induced toxicity in Nile tilapia.

### 2.3 Experimental Fish and Acclimation

A total of 240 healthy Nile tilapia (*Oreochromis niloticus*) was obtained from the Sharnalata Agro Fisheries Ltd., Mymensingh, Bangladesh. Before the starting of the experiment, fish were acclimated in a 1500 L cemented tank for 15 days under optimal laboratory conditions (i.e., ambient temperature and photoperiods). Proper siphoning and aeration through air pumps were maintained in the tank to facilitate acclimation and minimize transportation stress on fingerlings. During this period, fish were fed a commercial diet at a rate 3%–5% of body weight twice daily at 9 AM and 6 PM. During the acclimation and the experimental period, water quality parameters were monitored and maintained within ranges suitable for Nile tilapia.

### 2.4 Experimental Design

The exposure trial was conducted in 12 glass aquaria (75×45×45 cm^3^; nominal volume 100 L) at the Laboratory of Fish Ecophysiology, Bangladesh Agricultural University, Mymensingh. Each aquarium was stocked with 20 fingerlings for 42 days. Four experimental treatments, each with three replications, were maintained, such as the control group with no test chemical/additives, Hg‐exposed group with mercury (0.03 mg/L), Hg + Se group with mercury (0.03 mg/L) and selenium (3 mg/kg feed), and Hg + Vit‐E supplemented group with mercury (0.03 mg/L) and vitamin E (100 mg/kg feed). All the treatments received the same commercial diet with or without mended with test chemicals/additives. The fish were fed twice daily, in the morning at 9 AM and in the afternoon at 6 PM. After feeding, fish were allowed 1 h to consume feed, and then uneaten feed and debris were siphoned from tanks. Continuous aeration was provided in each tank using air pump (SOBO, Model: SB‐348A) to maintain adequate dissolved oxygen level. The regular measurements of temperature, pH, and ammonia were conducted using a mercury thermometer, a portable pH meter (RI 02895, HANNA Instruments Co., Dhaka, Bangladesh) and an API ammonia test kit (Mars Fishcare North America Inc., 50 E Hamilton St., Chalfont, PA 18914, USA). At the end of the exposure period (Day 42), fish were sampled, and growth, haematobiochemical profiles and histopathological alterations were assessed.

### 2.5 Growth Performance and Feed Utilisation

All the fish were carefully weighed to obtain their final body weight. Growth performances and feed efficiency were evaluated by measuring parameters such as WG, SGR, feed conversion ratio (FCR) and survival rate. The metrics were calculated using standard formulas to provide a comprehensive assessment of growth and nutrient utilisation.

$$\mathrm{Weight}\;\mathrm{gain}\;\left(g\right)=\mathrm{Final}\;\mathrm{weight}\;\left(\mathrm{gm}\right)\;-\;\mathrm{Initial}\;\mathrm{weight}\;\left(\mathrm{gm}\right)$$

$$\mathrm{SGR}\;\left(\%/\mathrm{day}\right)=\frac{\mathrm{In}\;\left(\mathrm{FBW}\right)-\;\mathrm{In}\;\left(\mathrm{IBW}\right)\;}{\mathrm{Number}\;\mathrm{of}\;\mathrm{days}\;}\;\;\times 100$$

$$\mathrm{FCR}\;=\frac{\mathrm{Feed}\;\mathrm{given}\;\left(\mathrm{dry}\;\mathrm{weight}\right)}{\mathrm{Body}\;\mathrm{weight}\;\mathrm{gain}\;\left(\mathrm{wet}\;\mathrm{weight}\right)}$$

$$\begin{array}{ccc} \mathrm{Survival}\;\left(\%\right) & = & \frac{\mathrm{Number}\;\mathrm{of}\;\mathrm{fish}\;\mathrm{survived}\;\mathrm{at}\;\mathrm{the}\;\mathrm{end}\;\mathrm{of}\;\mathrm{experiment}}{\mathrm{Initial}\;\mathrm{number}\;\mathrm{of}\;\mathrm{fish}\;\mathrm{stocked}\;}\\ & & \times \;100 \end{array}$$

### 2.6 Sampling and Tissue Collection

Feeding was stopped for 24 h before sampling. To reduce movement as well as stress, fish were anesthetised using clove oil (5 mg/L). A 5 µl of blood was drawn from the caudal region of the fish and poured into the Eppendorf tube, which contains the same number of anticoagulants (EDTA‐PBS buffer, pH 6.8–7) for smearing. After removing the fat and blood content from the intestines and gills, they are fixed in the Bouin's fluid for 24 h, followed by 70% alcohol for histological procedures.

### 2.7 Measurement of Haemato‐Biochemical Indices

Six (*n = *6) fishes from each tank were randomly sacrificed after the fishes had been raised for 42 days, and blood was drawn from the caudal region using a micropipette. A digital EasyMate GHb blood glucose/haemoglobin dual‐function monitoring system (Model: ET‐232, Bioptik Technology Inc., Taiwan 3505 7) was put to use to determine haemoglobin (g/dL) and glucose (mg/dL) using strips.

### 2.8 Assessment of Cellular and Nuclear Anomalies in Erythrocytes

A standard scientific protocol was employed to identify different types of erythrocytic cellular (ECA) and nuclear abnormalities (Shahjahan et al. 2019). Blood smears were prepared on thin glass slides, followed by air drying for 15 min. Pure ethyl alcohol was used to fix the smear. After air‐dried, staining was accomplished with 5% Giemsa stain for 10 mins. The stained smears were seen under a light microscope at 40X zoom (Micros, MCX100, Austria). Two blood slides were selected from each fish. The pictures were taken from a light‐dense area with approximately 500 cells, with 5 pictures from each slide. The ECA and erythrocytic nuclear abnormalities (ENA) were identified and converted into frequency (%).

### 2.9 Histopathological Analysis of Intestine and Gill

For histological procedures, the fixed intestine and gill samples were introduced into the next steps. Samples were dehydrated through an elevating series of alcohol (80%, 95%, 100% and 100%). Then clearing was executed through xylene followed by infiltration and embedding in the block with paraffin wax. 5 µm sections were prepared from the blocks. Glass slides containing the sections were deparaffinised, rehydrated in a diminishing series of alcohol (100%, 100%, 80% and 70%), and stained using Mayer's haematoxylin and eosin (H&E) stains. To visualise and enumerate the goblet cells, alcian blue stain was utilised. Both quantitative and semi‐quantitative analyses were performed on the histological sections. Measurement of villus length, villus width, crypt depth, thickness of muscularis, fattening of mucosal fold and width of lamina propria were determined from the intestines of each treatment group. In the gills, clubbing in the secondary lamellae, congestion in the primary lamellae, hypertrophy and hyperplasia in the primary lamellae, hypertrophy in the secondary lamellae and degeneration in the secondary lamellae were analysed. In addition, intestinal and gill deformities were identified using 20–40 slides per treatment. The percentage of deformed slides for each abnormality was determined by dividing the number of slides exhibiting the abnormality by the total number examined (*n = *20) and multiplying by 100. The severity of lesions was categorised as follows: ‘‐’ = 0% (no occurrence), ‘+’ = < %10% (mild), ‘++’ = 10%–50% (moderate) and ‘+++’ = > 50% (severe) (Shahriar et al. 2024).

## 3 Results

### 3.1 Growth Performances

Growth and feed conversion performances of fish cultured in different treatments are summarised in Table 1. The study found significantly (*p < *0.05) the lowest final body weight (FBW, 24.96 ± 4.44 g) and weight gain (WG, 14.14 ± 4.44 g) in fish cultured in Hg group; however, supplementation with either vitamin E or Selenium nanoparticles significantly enhanced these parameters compared to Hg, even with control group. Besides, Hg group exhibits significantly the slowest (*p < *0.05) specific growth rate (SGR, 0.85 ± 0.18 %/day) and highest feed conversion ratio (FCR) among the treatments, but vitamin E and selenium supplement significantly increased SGR and reduced FCR compared to Hg group but no significant differences with the control. In the case of survival, Hg group experienced significantly lower (*p < *0.05) compared to any treatments experimented (Table 1).

**TABLE 1:** Growth and feed utilization performances in Nile tilapia, *Oreochromis niloticus*.

Parameters	Treatments			
	Control	Hg	Hg + Vit‐E	Hg + Se‐NP
IBW (g)	10.82 ± 1.05^a^	10.82 ± 1.05^a^	10.82 ± 1.05^a^	10.82 ± 1.05^a^
FBW (g)	30.72 ± 5.55^b^	24.96 ± 4.44^a^	32.90 ± 3.60^bc^	34.44 ± 5.74^c^
WG (g)	19.90 ± 5.55^b^	14.14 ± 4.44^a^	22.08 ± 3.60^bc^	23.62 ± 5.74^c^
SGR (%/day)	1.06 ± 0.17^b^	0.85 ± 0.18^a^	1.14 ± 0.11^b^	1.18 ± 0.18^b^
FCR	1.18 ± 0.29^a^	1.73 ± 0.68^b^	1.02 ± 0.17^a^	1.00 ± 0.29^a^
Survival (%)	100 ± 0.00^b^	87.5 ± 2.74^a^	100 ± 0.00^b^	100 ± 0.00^b^

*Note*: Superscripts (a, b, c and bc) denote significant variance (p <0.05) between treatments.

Abbreviations: FBW, final body weight; FCR, feed conversion ration; Hg, mercury; IBW, initial body weight; Se‐NP, selenium nano particles; SGR, specific growth rate; Vit‐E, vitamin E; WG, weight gain.

### 3.2 Metabolic Markers

Status of systemic stress markers (i.e., haemoglobin and glucose) are summarised in Figure 1A,B. Exposure to mercury (Hg) significantly (*p < *0.05) increased haemoglobin and glucose levels; however, vitamin E + Hg and Hg + Se co‐supplemented groups significantly reduced this increment compared to Hg group and produced non‐significant differences with the control group (Figure 1).

**FIGURE 1:**
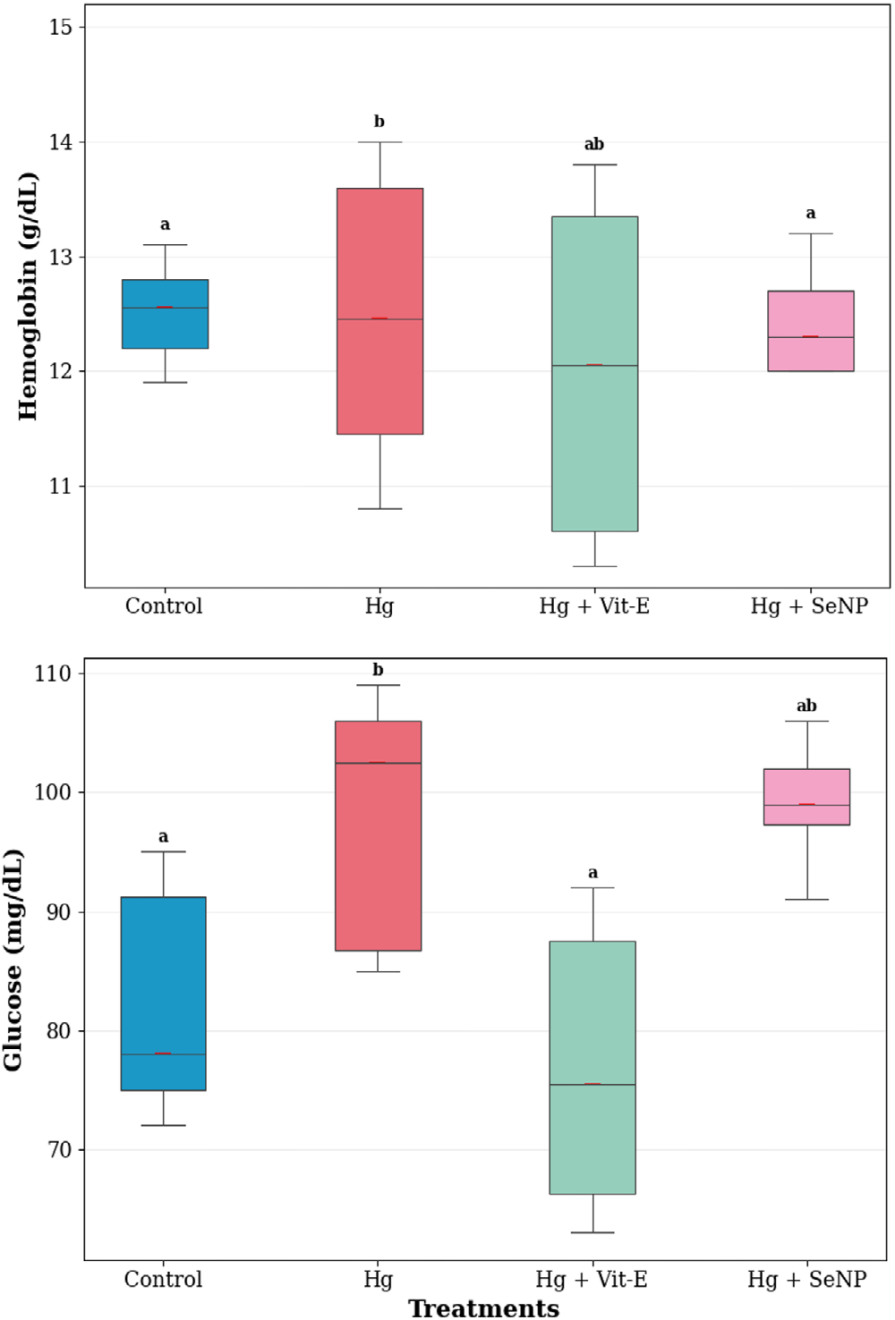
Level of blood haemoglobin (A) and glucose (B) in fish reared in different treatments such as control (no test chemical/additives), mercury (Hg; 0.03 mg/L), Hg + Se (Hg; 0.03 mg/L, and selenium; 3 mg/kg feed) and Hg + Vit‐E (Hg; 0.03 mg/L, and vitamin E; 100 mg/kg feed). The different alphabets in the superscripts indicate significant differences among treatments (*p* < 0.05).

### 3.3 Erythrocyte Cellular and Nuclear Morphology

Frequencies of ECA and ENA abnormalities of fish reared in different regimes are depicted in Figure 2A,B. Twin‐shaped cells increased significantly in Hg‐exposed fish compared to control; however, vitamin E supplements significantly restored the damage compared to Hg group but not with the control. But selenium was still found significantly higher in twins compared to control suggesting ineffective restoration. Fusion and teardrop‐type cells also increased significantly in Hg‐exposed group compared to control, but relatively significant restoration in vitamin E or selenium co‐exposed groups. In addition, frequency of elongated cells was found significantly highest in Hg‐exposed group, significantly declined in both the supplemented group, but still remains significantly higher than the control fishes (Figure 2A).

**FIGURE 2:**
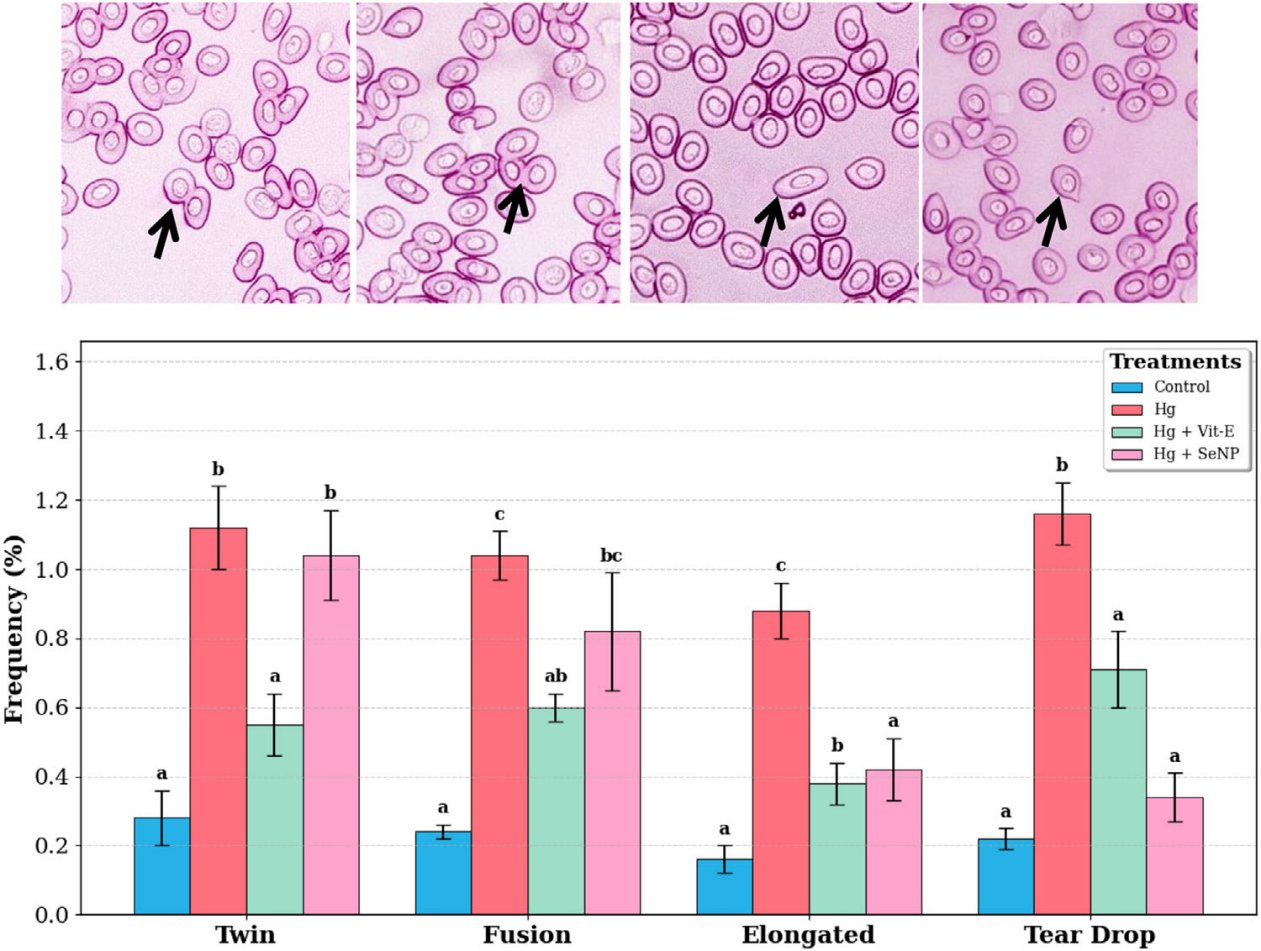
Frequencies of erythrocyte cellular abnormalities (ECA) in fish reared in different treatments such as control (no test chemical/additives), mercury (Hg; 0.03 mg/L), Hg + Se (Hg; 0.03 mg/L, and selenium; 3 mg/kg feed) and Hg + Vit‐E (Hg; 0.03 mg/L, and vitamin E; 100 mg/kg feed). The different alphabets in the superscripts indicate significant differences among treatments (*p* < 0.05).

Besides, frequencies of nuclear abnormalities (ENAs) are also changed in Hg‐exposed group and Hg‐plus‐supplemented groups (Figure 3). Frequencies of notched nuclei and nuclear buds increased significantly in Hg group from control and both the supplements (Vit E & SeNP) restored the nuclear damage significantly and bring back the values close to control group. More or less the same pattern of changes observed in karyopyknotic nuclei; increased occurrence in Hg‐exposed group but significant attenuation was recorded in the latter two co‐exposed groups. These curative findings of both erythrocyte cellular and nuclear anomalies through vitamin E and selenium suggest significant protective effects of these supplements on blood morphological indices.

**FIGURE 3:**
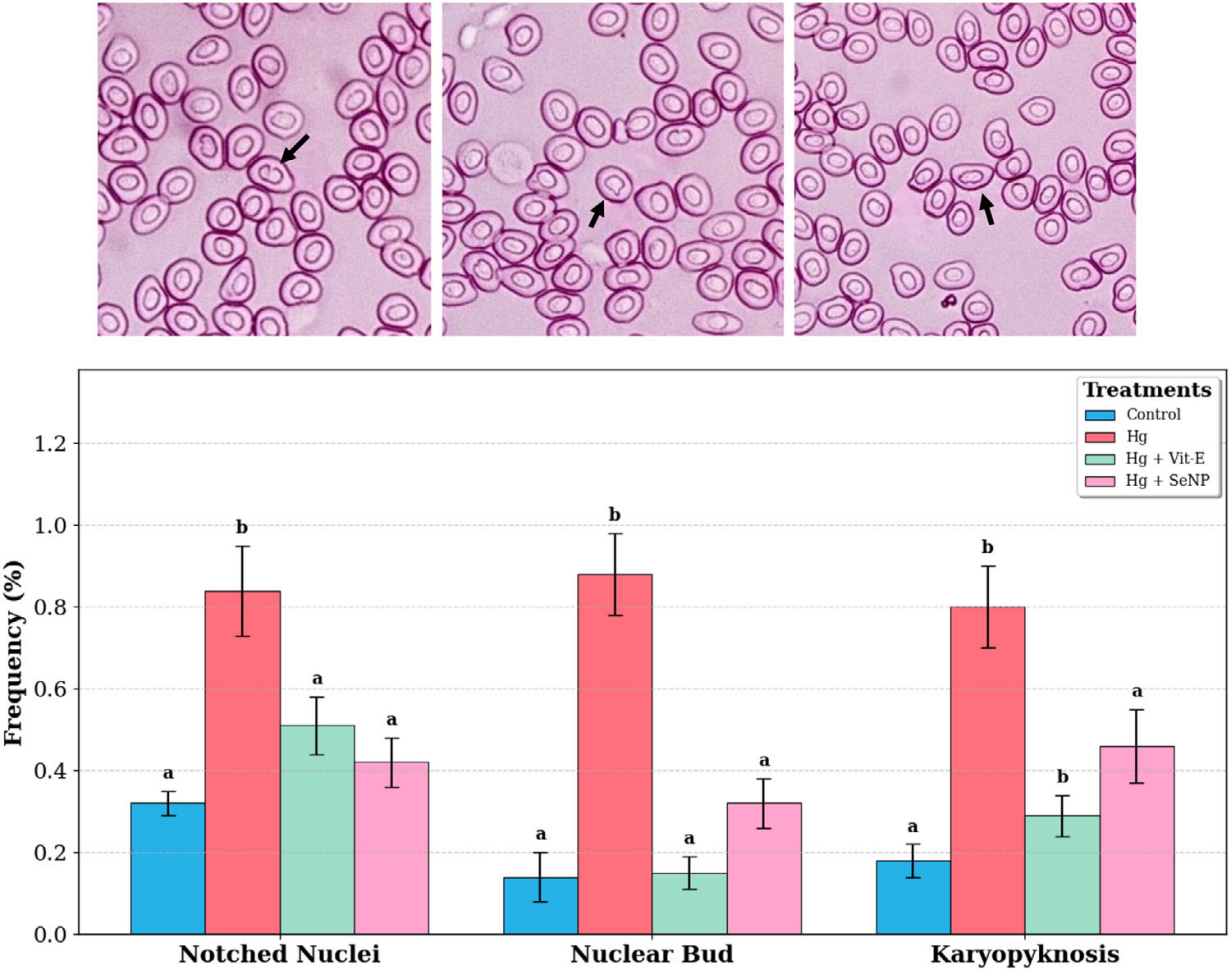
Frequencies of erythrocyte nuclear abnormalities (ENA) in fish reared in different treatments such as control (no test chemical/additives), mercury (Hg; 0.03 mg/L), Hg + Se (Hg; 0.03 mg/L, and selenium; 3 mg/kg feed) and Hg + Vit‐E (Hg; 0.03 mg/L, and vitamin E; 100 mg/kg feed). The different alphabets in the superscripts indicate significant differences among treatments (*p* < 0.05).

### 3.4 Histopathology of Gills

Semi‐quantitative assessment of gill deformities and protection by supplements (Figure 4) are summarised in Table 2. Congestion in primary lamellae and clubbing in the secondary lamellae appeared in Hg group with moderate severity; still observed in vitamin E‐supplemented group with mild changes in both primary and secondary lamellae; however, clubbing was completely cured in selenium added group. Besides, hypertrophy in primary and secondary lamellae occurred in Hg‐exposed fishes with moderate and severe grade, respectively and was restored in supplemented co‐exposed group with null to mild changes. Similarly, degeneration of secondary lamellae was most pronounced in mercury‐exposed group with severe changes, but incomplete protection was observed in vitamin E compared to SeNP (Table 2). Besides the semiquantitative assessment, morphological changes in gill and additive‐instigated protection are presented in Figure 4.

**FIGURE 4:**
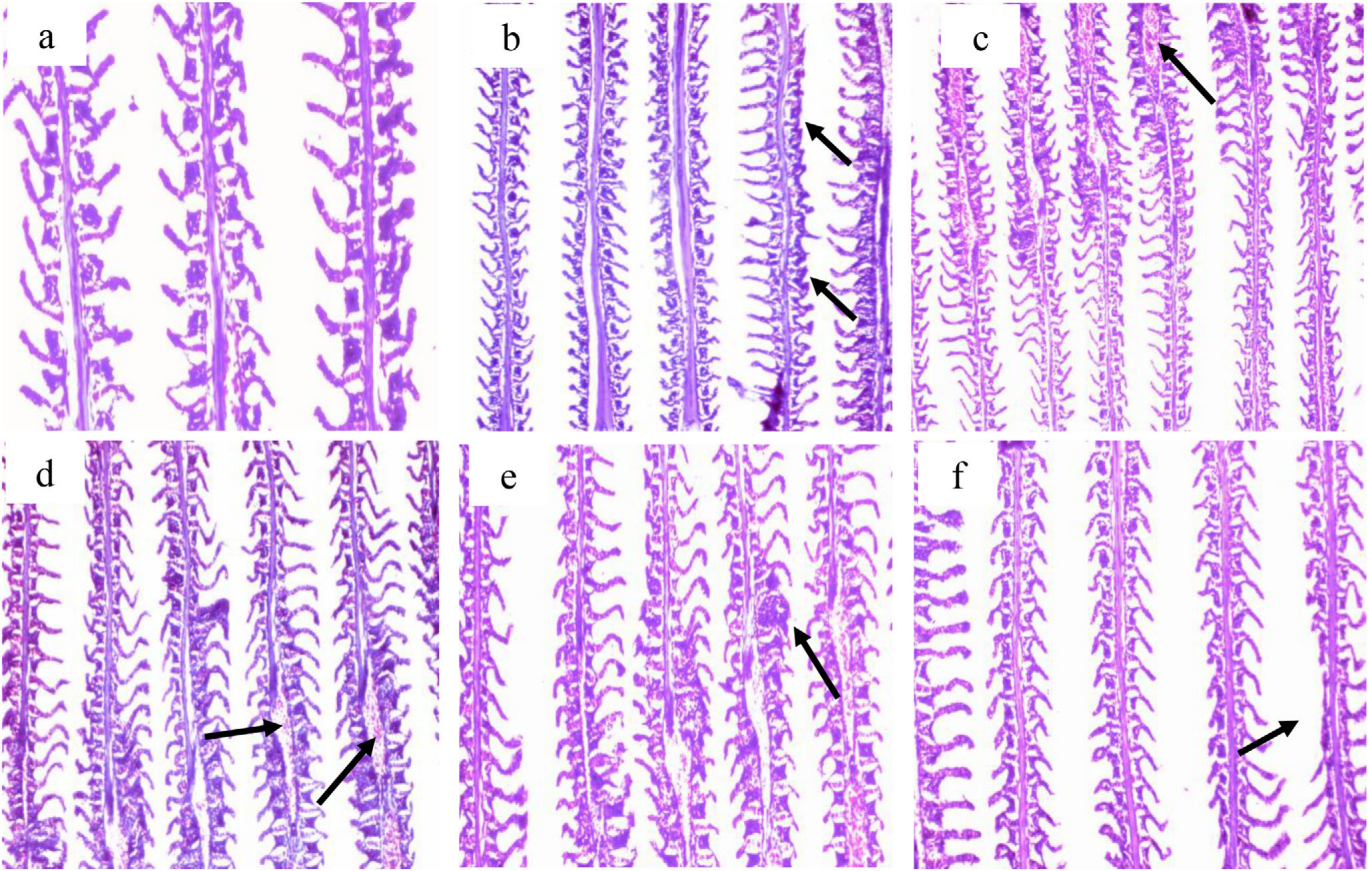
Histomorphological changes in gills of tilapia exposed to different treatments such as control (no test chemical/additives), mercury (Hg; 0.03 mg/L), Hg + Se (Hg; 0.03 mg/L, and selenium; 3 mg/kg feed) and Hg + Vit‐E (Hg; 0.03 mg/L, and vitamin E; 100 mg/kg feed). In figures: a, normal gills; b, clubbing in the secondary lamellae; c, congestion in the primary lamellae; d, hypertrophy and hyperplasia in the primary lamellae; e, hypertrophy in the secondary lamellae; f, degeneration in the secondary lamellae.

**TABLE 2:** Gill deformities and restorations in Nile tilapia, *Oreochromis niloticus*.

Tissue abnormalities	Treatments			
	Control	Hg	Hg + Vit‐E	Hg + Se‐NP
Clubbing in the secondary lamellae	—	++	+	—
Congestion in the primary lamellae	—	++	+	+
Hypertrophy and hyperplasia in the primary lamellae	—	++	—	—
Hypertrophy in the secondary lamellae	—	+++	+	+
Degeneration in the secondary lamellae	—	+++	++	—

*Note*: No abnormalities (−, 0%), mild abnormalities (+, 10%), moderate abnormalities (++,10–50%) and severe abnormalities (+++, >50%).

Abbreviations: Hg, mercury; Se‐NP, selenium nano particles; Vit‐E, vitamin E.

### 3.5 Histopathology of Intestine

Assessment of changes in intestinal morphology and pathology (Figure 5) are summarised in Table 3. Mercury exposure significantly reduced villus length, wall thickness, muscle thickness, crypt depth, mucosal fold fattening and goblet cell abundance, while increasing lamina propria width compared with the control group (*p < *0.05). However, villus width was highest in Hg group and lowest in Hg + SeNP group. In the case of another supplement, vitamin E restored villus length, wall and muscle thickness to values not significantly different from the control, and partially improved crypt depth and fattening of mucosal fold. However, selenium nanoparticle supplements significantly improved crypt depth and recovered structural thickness of the wall and muscularis, but villus length remained similar to the Hg group. Both supplements normalised lamina propria width relative to the Hg group. Overall, these findings indicate both SeNP and vitamin E as effective agents in attenuating mercury‐induced intestinal damage in tilapia (Table 3). In addition, frequency of goblet cells decreased significantly in Hg group from control and significant restoration was recorded in both vitamin E and selenium co‐exposed groups (Figure 6).

**FIGURE 5:**
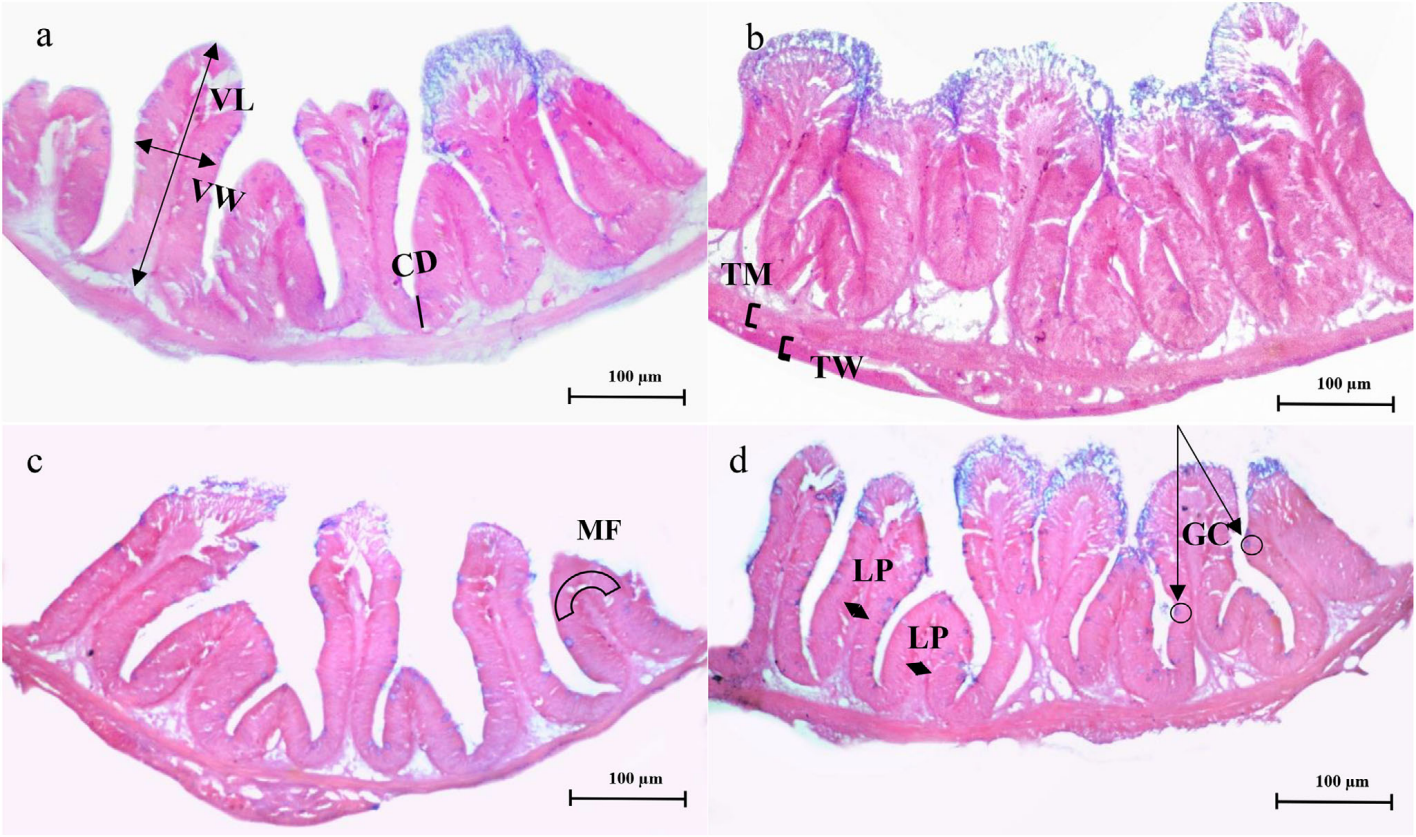
Histomorphological changes in the intestine of tilapia exposed to different treatments such as control (no test chemical/additives), mercury (Hg; 0.03 mg/L), Hg + Se (Hg; 0.03 mg/L, and selenium; 3 mg/kg feed) and Hg + Vit‐E (Hg; 0.03 mg/L, and vitamin E; 100 mg/kg feed).

**TABLE 3:** Histomorphological changes in intestine of Nile tilapia, *Oreochromis niloticus*.

Parameters	Treatments			
	Control	Hg	Hg + Vit‐E	Hg + Se‐NP
Villus length (µm)	280.04 ± 9.79^b^	258.91 ± 9.42^a^	276.15 ± 7.96^b^	260.31 ± 13.69^a^
Villus width (µm)	85.89 ± 7.69^bc^	90.37 ± 4.97^c^	82.78 ± 3.57^b^	67.52 ± 5.40a
Thickness of the wall (µm)	16.73 ± 1.32^b^	6.74 ± 0.94^a^	17.05 ± 1.68^b^	15.79 ± 2.47^b^
Thickness of muscular (µm)	23.38 ± 1.45^b^	14.31 ± 1.45^a^	23.77 ± 2.28^b^	24.06 ± 3.93^b^
Crypt depth (µm)	56.67 ± 7.81^b^	38.31 ± 2.85^a^	63.96 ± 5.94^b^	79.30 ± 5.62^c^
Fattening of mucosal fold (µm)	112.09 ± 4.57^c^	84.56 ± 2.79^a^	101.88 ± 7.63^b^	99.64 ± 5.21^b^
Width of lamina propria (µm)	14.64 ± 1.89^a^	19.31 ± 1.09^b^	13.82 ± 3.25^a^	13.00 ± 2.34^a^

*Note*: Superscripts (a, b and c) denote significant variance (*p < *0.05) between treatments.

Abbreviations: Hg, mercury; Se‐NP, selenium nanoparticles; Vit‐E, vitamin E.

**FIGURE 6:**
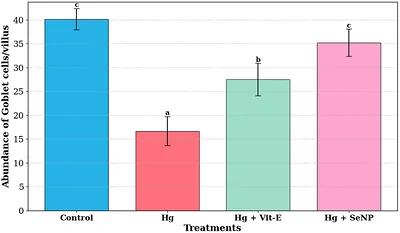
Frequency of occurrences of goblet cells in intestine of Nile tilapia exposed to different treatments such as control (no test chemical/additives), mercury (Hg; 0.03 mg/L), Hg + Se (Hg; 0.03 mg/L, and selenium; 3 mg/kg feed) and Hg + Vit‐E (Hg; 0.03 mg/L, and vitamin E; 100 mg/kg feed). The different alphabets in the superscripts indicate significant differences among treatments (*p* < 0.05).

### 3.6 Principal Component Analysis (PCA)

The principal component analysis (PCA) biplot demonstrates the relationship between several treatments and parameters based on their contributions to the two principal components (PCs) are presented in Figure 7. Treatment groups clustered separately in the PCA, which explained 93.4% of the variance (PC1: 75.3%; PC2: 18.1%), which indicates varied biological effects of experimental exposure (Figure 7). The Hg‐treated group clustered along the negative PC1 axis, correlating with stress‐related traits; however, the control group aligned positively with growth and condition variables. Notably, both supplemented groups shifted closer to the control cluster, with SeNP showing greater restoration of growth traits. These patterns suggest that SeNP co‐treatment provides more effective mitigation of mercury‑induced physiological disturbances.

**FIGURE 7:**
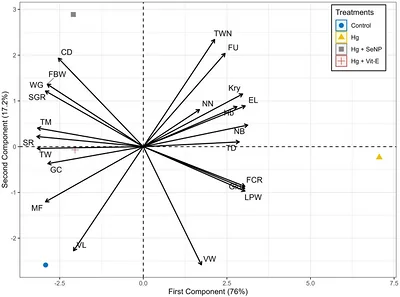
Principal component analysis biplot showing the growth, haemato‐biochemical parameters, histological changes of tilapia exposed to different treatments such as control (no test chemical/additives), mercury (Hg; 0.03 mg/L), Hg + Se (Hg; 0.03 mg/L, and selenium; 3 mg/kg feed) and Hg + Vit‐E (Hg; 0.03 mg/L, and vitamin E; 100 mg/kg feed). In figure: CD, crypt depth; EL, elongated; FBW, final body weight; FCR, feed conversion ratio; FU, fusion; GC, goblet cells; Glu, glucose; Hb, haemoglobin; Kry, karyopyknosis; LPW, width of lamina propria, MF, fattening of mucosal fold; NB, nuclear bud; NN, notched nuclei; SGR, specific growth rate, SR, survival rate, TD, tear drop; TM, thickness of intestinal muscular layer; TW, thickness of intestinal wall; TWN, twin; VL, villus length; VW, villus width; WG, weight gain.

## 4 Discussion

The present study clearly demonstrates that sublethal mercury exposure produces pronounced impairments in growth performances, blood biochemical index, erythrocyte integrity and gill histology in Nile tilapia, and that dietary supplementation by vitamin E or selenium nanoparticles (SeNP) significantly attenuated most of the effects.

Growth performances and feed conversion to biomass were suppressed by mercury and were restored by both vitamin E or SeNP additives. Reduced final body weight, weight gain and specific growth rate and increased FCR following mercury exposure are found consistent with previous reports that mercury impairs nutrient assimilation and metabolic performances in fishes (Canham et al. 2020). Specific to tilapia, dietary inorganic mercury and methyl mercury both hindered growth performances (Wang and Wang 2012; Alam et al. 2021; Mahboub et al. 2021). However, the growth and feed utilisation metrics in SeNP and vitamin E supplemented in tilapia also align with experimental evidence that vitamin E protects growth by preserving membrane integrity and digestive function under oxidative stress (Dawit Moges et al. 2022; El‐Sayed and Izquierdo 2022). Study reports that vitamin E prevents phospholipid peroxidation of cellular and subcellular membrane and red blood cells, and helps to maintain functional and structural integrity of fish cells, and improve immune response, enhance growth and feed use efficacy, prevent muscle loss and improve overall biological productivity of fish, even vitamin E can interact with other micronutrients including selenium (El‐Sayed and Izquierdo 2022). Dietary SeNP likely improved the growth by enhancing intestinal integrity and digestive efficiency and augmenting the activity of selenoenzymes that reduce systemic oxidative burden; improved gut morphology and functions of enzymes increase nutrient adsorption and conversion to biomass, thereby reversing mercury‐caused growth inhibition (Al‐Wakeel et al. 2024). Besides, vitamin E supports growth increment possibly by protecting enterocyte membrane and lipids from peroxidation, protecting membrane fluidity and absorptive surface functions during oxidative challenges (El‐Sayed and Izquierdo 2022). The improved growth performance in the additive groups may be associated with reduced metabolic stress and better nutrient assimilation under Hg stress condition. However, Hg‐induced oxidative stress can disturb cellular metabolism and increase energy requirements for stress responses, which may impair growth in turn. Study suggests that dietary SeNP and vitamin E can improve growth performance and FCR that closely align with the current findings (Dawood et al. 2020). Moreover, selenium is an essential component of several antioxidant selenoproteins, especially glutathione peroxidase, and vitamin E acts like fat‐soluble antioxidant that limits lipid peroxidation and helps to maintain integrity of the membrane (Dawood et al. 2020). However, reports suggest an additional mechanism may involve in the antagonistic interaction between selenium and mercury. Selenium can interact with Hg and form relatively stable Hg‐Se compounds, potentially reducing the biological reactivity of Hg and helping preserve selenium for selenoprotein‐dependent antioxidant functions (Adams and Duguay 2025).

Observed hematobiochemical parameters in the current study, notably elevations in blood glucose and in haemoglobin are characteristic stress response to metal exposure and reflect mobilisation of energy reserves and impaired oxygen transport capacity reported in other fish species exposed to mercury (Zulkipli et al. 2021). Concurrent surge in glucose and haemoglobin probably due to acute stress and osmoregulatory disturbances by mercury (HgCl_2_) that might have triggered spleen contraction and haemoconcentration (release of stored erythrocytes and resulting Hb surge) (Wendelaar Bonga 1997) while activating neuroendocrine stress pathways that raised glucose concentration (Handayani et al. 2020). Exposure to dietary mercury (0.5 mg/kg feed) caused significant variation in Hb, thrombocytes (Trb), MCH, MCHC, MN and ENAs in tilapia *Oreochromis sp*. in previous study aligning with our results (Liscano‐Carreño et al. 2025). The restoration of these indices by vitamin E and selenium nanoparticles indicates mitigations of systemic stress and improved physiological homeostasis. This protective effect likely arises from restoration of redox condition and stabilisation of erythrocyte membranes by lipid‐soluble antioxidants and selenium‐dependent selenoenzymes that reduce oxidative damage in fishes (Cabezas‐Sanchez et al. 2019). Moreover, mercury induced marked changes in ECA and ENA in fish of current investigation. Such cytological and genetic endpoints are indicative of mercury‐induced DNA damage and membrane damage in fish and also have been associated with oxidative stress and direct genotoxic actions of metals (Shahjahan et al. 2019). Significant mitigation of erythrocytic anomalies in supplemented co‐exposed groups suggests a mechanistic role of antioxidant protection in preserving nuclear integrity. Vitamin E likely limits peroxidative damage to membrane lipids and sialoproteins that might detoxify peroxides and repair oxidative lesions (Tocher et al. 2002).

In the present study, histopathological assessment revealed typical mercury‐induced lesions in gills and intestine such as epithelial hyperplasia, lamellar degeneration, villus atrophy, thinning of the mucosal wall and loss of goblet cells. These morphological and pathological alterations describe mechanistic insights for functional impairments in growth and blood profiles. Short‐term exposures to mercury to tilapia have been documented to cause microscopic lesions in the kidney, hepatopancreas, spleen and intestine (Kaewamatawong et al. 2013), and chronic exposure caused disintegration of gill epithelial cells, desquamated epithelium, haemorrhaged and complete damage of epithelial lamellar cells (Chezhian et al. 2012). Comparable spectra of lesions have also been reported in mercury‐exposed fishes (Canham et al. 2020). In our trial, both vitamin E and SeNP substantially mitigated lesion severity, although the pattern of protection differed by supplement and parameter type. Vitamin E was found more effective in preservation of villus length, wall and muscularis thickening, consistent with its role in membrane protection and maintenance of enterocyte integrity (Dawit Moges et al. 2022). SeNP produced marked increases in crypt depth and promoted recovery of goblet cell abundance, effects that may reflect enhanced mucosal regeneration and improved mucous barrier function afforded by selenium, instigated redox‐sensitive repair pathways and immunomodulatory effects (Dawit Moges et al. 2022). In addition, the observed improved histological condition of tissues in the supplemented groups may be related to reduced oxidative damage and preservation of cellular and membrane structure. In that case, selenium‐dependent peroxidases may be involved in the removal of hydrogen peroxide and lipid hydroperoxides, while vitamin E can directly inhibit lipid peroxidation within biological membranes (Wang and Quinn 1999).

Taken together, the data support a mechanistic cascade in which mercury impairs growth primarily by disruption of intestinal structure and through systemic stress affecting blood physiology. However, vitamin E appears to stabilize membrane integrity of lipids and preserve existing tissue architecture. Besides, selenium nanoparticles may enhance epithelial regeneration and mucosal defence, likely via improved selenium bioavailability that repairs processes and selenoprotein function and reduction of bioavailability of mercury through chemical complexes. Therefore, we assume the combined outcomes yielded complementary protection of absorptive capacity and organismal homeostasis (Dawit Moges et al. 2022; Moniruzzaman et al. 2017).

## 5 Conclusion

The present study describes how sublethal mercury exposures produced significant hematobiochemical and histopathological insults in Nile tilapia that together explain reduced growth, poor feed conversion and physiological stress. Dietary vitamin E and SeNP substantially mitigated these outcomes. It is indicative that the complementary protective effects of both the additives significantly restored physiological homeostasis and provided the best growth and feed utilisation. We sincerely acknowledge the limitations of the current study such as lack of analytical verification of concentrations of chemicals in water, measurement of Hg in fish tissue including uptake and elimination for better evidence of complete remediation, due to limited resources. However, we are planning to collaborate with analytical chemistry laboratory for measurement of our samples in future studies. This study recommends synthesis of tissue concentration of both toxins and additives used and toxicokinetic assessment for better understanding of portioning and distribution of metals in fish body.

## Author Contributions

**Mir Ashrafia Ananna**: writing – original draft, investigation, formal analysis, data curation. **Abhijit Dash**: writing – original draft, investigation, formal analysis, data curation. **AKM Afzal Hossain**: investigation, formal analysis, data curation. **Md Ruhul Amin**: formal analysis. **Md Shahjahan**: writing – review and editing, supervision, funding acquisition, conceptualization. **Md Al‐Emran**: writing – review and editing, supervision.

## Funding

The authors have nothing to report.

## Ethics Statement

All the experimental procedures were performed in accordance with the Animal and Ethical Committee of Bangladesh Agricultural University, Mymensingh‐2202, Bangladesh (Approval No. BAU‐FoF/2025/002). Efforts were made to minimize animal sufferings and to use the minimum number of animals required to obtain reliable results.

## Conflicts of Interest

The authors declare no conflicts of interest.

## Data Availability

Data will be available on reasonable request to corresponding author.
